# One Universal Common Endpoint in Mouse Models of Amyotrophic Lateral Sclerosis

**DOI:** 10.1371/journal.pone.0020582

**Published:** 2011-06-08

**Authors:** Jesse A. Solomon, Mark A. Tarnopolsky, Mazen J. Hamadeh

**Affiliations:** 1 School of Kinesiology and Health Science, Faculty of Health, York University, Toronto, Ontario, Canada; 2 Department of Pediatrics, McMaster University, Hamilton, Ontario, Canada; 3 Department of Medicine, McMaster University, Hamilton, Ontario, Canada; Johns Hopkins, United States of America

## Abstract

There is no consensus among research laboratories around the world on the criteria that define endpoint in studies involving rodent models of amyotrophic lateral sclerosis (ALS). Data from 4 nutrition intervention studies using 162 G93A mice, a model of ALS, were analyzed to determine if differences exist between the following endpoint criteria: CS 4 (functional paralysis of both hindlimbs), CS 4+ (CS 4 in addition to the earliest age of body weight loss, body condition deterioration or righting reflex), and CS 5 (CS 4 plus righting reflex >20 s). The age (d; mean ± SD) at which mice reached endpoint was recorded as the unit of measurement. Mice reached CS 4 at 123.9±10.3 d, CS 4+ at 126.6±9.8 d and CS 5 at 127.6±9.8 d, all significantly different from each other (P<0.001). There was a significant positive correlation between CS 4 and CS 5 (r = 0.95, P<0.001), CS 4 and CS 4+ (r = 0.96, P<0.001), and CS 4+ and CS 5 (r = 0.98, P<0.001), with the Bland-Altman plot showing an acceptable bias between all endpoints. Logrank tests showed that mice reached CS 4 24% and 34% faster than CS 4+ (P = 0.046) and CS 5 (P = 0.006), respectively. Adopting CS 4 as endpoint would spare a mouse an average of 4 days (P<0.001) from further neuromuscular disability and poor quality of life compared to CS 5. Alternatively, CS 5 provides information regarding proprioception and severe motor neuron death, both could be important parameters in establishing the efficacy of specific treatments. Converging ethics and discovery, would adopting CS 4 as endpoint compromise the acquisition of insight about the effects of interventions in animal models of ALS?

## Introduction

The endpoints used in research studies involving mouse models of amyotrophic lateral sclerosis (ALS) vary widely between laboratories around the world [1]–[97]. Not all endpoints are in perfect agreement with each other; that is, depending on the endpoint, the age at which mice are euthanized (time point at which age is used to establish lifespan) may differ independently of the intervention treatment used in research studies (Table 1). Because specific endpoints inherently occur early or later in disease progression, researchers may not be able to directly compare the effectiveness of treatments on disease severity and progression, as well as functional outcomes, in animal models of ALS conducted in one research laboratory with similar treatments conducted in different laboratories. Identifying the proper endpoint is important, because it impacts lifespan and marks the end of data collection. A solution would be a universal endpoint which would allow researchers to follow disease progression and identify the effectiveness of an intervention treatment, while still meeting stringent ethical standards. Of relevance, this impacts the adoption of results from animal-based research for human randomized clinical trials, as well as the implications of adopting new recommendations, nutrition or pharmaceutical, for people with ALS.

**Table 1:** Raw data and summary of endpoint criteria for 162 B6SJL-TgN-(SOD1-G93A)1Gur autosomal hemizygous female (F) and male (M) mice.

Endpoint Criteria	N Total (F, M)	% of Mice Meeting Criteria (F, M)	Mean Age (d) (F, M)
CS 4	162 (100, 62)	100.0% (100.0%, 100.0%)	123.9±10.3* (125.8±10.7, 120.8±8.8)
20%CS2	47 (28, 19)	29.0% (28.0%, 30.6%)	128.1±10.7 (129.5±12.1, 125.9±8.5)
20%Peak	69 (40, 29)	42.6% (40.0%, 46.8%)	126.6±10.8 (128.9±11.6, 123.6±8.7)
BC<2	42 (16, 26)	25.9% (16.0%, 41.9%)	121.6±11.5 (127.8±8.3, 117.7±11.7)
CS 4+	162 (100, 62)	100.0% (100.0%, 100.0%)	126.6±9.8* (129.1±9.9, 122.5±8.3)
CS 5	162 (100, 62)	100.0% (100.0%, 100.0%)	127.6±9.8* (129.7±10.0, 124.1±8.5)

CS 4, clinical score of 4 = functional paralysis of both hindlimbs; 20%CS2, weight loss ≥20% *vs.* body weight immediately prior to a clinical score of 2; 20%Peak, weight loss ≥20% *vs.* peak body weight; BC<2, body condition score <2; CS 5, clinical score of 5 = CS 4 plus a righting reflex >20 s; CS4+, clinical score of 4+ = CS 4 in addition to the earliest of 20%CS2, 20%Peak, BC<2 or a righting reflex of >20 s.

*Significantly different from each other (P<0.001). Data for Mean Age (d) are presented as means ± SD.

ALS is a devastating neuromuscular disease characterized by death of motor neurons in the brain [98] and spinal cord [99]. Symptoms of ALS begin with muscle weakness, ultimately leading to paralysis and death [100]. The first mouse model used to study ALS was created by Gurney et al in 1994 [100] who discovered that a glycine to alanine substitution on the 93^rd^ position in the human Cu,Zn superoxide dismutase (Cu/Zn-SOD) gene produced the phenotype of ALS. Mice testing positive for this mutation begin to overtly exhibit signs of motor degeneration through a change in gait between 85–110 d of life [15], [82], [83]. As the disease progresses, the hindlimbs become paralyzed, paw grip strength and endurance deteriorate, bony structures become palpable due to severe muscle and tissue loss, mobility is limited, and an inability to groom and scavenge for food and water become apparent [20], [49], [54], [73], [82]. Researchers conducting intervention studies in mouse models of ALS monitor the above changes to track the effectiveness of their intervention, however at what point is it no longer ethical to keep these mice alive?

Research ethics committees and animal care organizations/agencies serve to maintain standards for the care and use of animals used in research, including transgenic mice used in models of ALS [101]. Standards of care include the selection of “endpoint” which is the point at which an experimental animal is killed humanely to terminate pain, distress and/or suffering [101]. Hence, research ethics committees and animal care organizations/agencies must consult with ALS researchers to decide on a case-by-case basis which endpoint is suitable for a specific intervention study, while maintaining compatibility with the objectives and the integrity of the research project. Different laboratories using mouse models of ALS have chosen varying endpoints, some reflecting more advanced stages of the disease, including the righting reflex (mice are placed on their sides and are euthanized if they cannot right themselves to sternum in 3–30 s, time chosen depends on the laboratory), an inability to splay the hindlimbs due to paralysis, a percentage decrease in motor performance or grip strength from initial values, an inability to obtain food or water, a defined percentage of body weight loss from peak weight, serious eye infection, an inability to self-groom, no spontaneous breathing or movement for a predetermined time with no response to pain, complete hindlimb paralysis, or combinations of two or more of these criteria. The most commonly used endpoint is a righting reflex of at least 3 s [1], [4], [5], [7], [10]–[15], [17]–[23], [25], [27], [28], [30]–[32], [36], [38]–[47], [49]–[51], [53], [57]–[59], [61], [62], [64], [65], [68], [70]–[72], [74], [77], [79]–[83], [84], [86]–[89], [91], [94]–[97], however some studies did not specify the length of time used as the cutoff for the righting reflex [26], [54], [60], [73], [78], [85], [90], [93]. The popularity of the righting reflex is possibly due to its relative simplicity, value as an indicator of proprioception deterioration [102], [103], and/or history as the first endpoint used in an intervention study in this particular disease model [79].

To date, a universal endpoint has not been established among researchers using rodent models of ALS. An ideal endpoint would meet strict ethical standards, could be adopted by all research laboratories, and would allow researchers to properly study the progression of ALS and the effectiveness of treatments tested. A consistent endpoint across research laboratories would reduce inter-laboratory variability that may be attributed at least partially to the selection of endpoint. Thus, our objective was to determine whether an earlier endpoint could replace the righting reflex, sparing mice undue suffering, while preserving the integrity of research in rodent models of ALS. To do this, we used the G93A transgenic mouse model of ALS to validate if functional paralysis in both hindlimbs (CS 4) could replace other later endpoints, including the righting reflex (CS 5).

## Materials and Methods

### Ethics Statement

The experimental protocols of all 4 studies followed the guidelines of the Canadian Council of Animal Care and were approved by the McMaster University Animal Research Ethics Board. All necessary steps were taken to minimize suffering and distress to the mice in the studies.

### Animals

Raw data for clinical score (CS), body condition and body weight were compiled from 4 previously published [15], [82], [83], [104], [105] nutrition intervention studies using a total of 162 B6SJL-TgN-(SOD1-G93A)1Gur autosomal hemizygous mice (100 females, 62 males) that reached endpoint at a clinical score of 5 (CS 5). All mice expressed the phenotype of ALS due to the G93A mutation in the SOD1 (Cu/Zn-SOD) gene. Raw data were used to determine the following endpoint criteria, with age (d) at which mice reached endpoint as the unit of measurement:

1. CS 4 = both hindlimbs are functionally paralyzed
2. CS 4+ = CS 4 plus the earliest time mice attained one of the following:
  - weight loss ≥20% *vs.* body weight immediately prior to a clinical score of 2 (CS 2 is considered disease onset) = 20%CS2
  - weight loss ≥20% *vs.* peak body weight = 20%Peak
  - body condition score <2 = BC<2
  - righting reflex >20 s (clinical score of 5) = CS 5
3. CS 5 = CS 4 plus a righting reflex >20 s (considered as the endpoint in the previous 4 studies)

### Body Weight and Body Condition

Body weights of mice in the 4 intervention studies were measured starting at age 35–40 d until mice reached CS 5. Body condition was assessed following a 5-point scale: 5 = obese mice, 4 = overconditioned mice (spine is a continuous column and the vertebrae are palpable only with firm pressure), 3 = well-conditioned mice (the vertebrae and dorsal pelvis are not prominent and are palpable with slight pressure), 2 = underconditioned mice (the segmentation of the vertebral column is evident and the dorsal pelvic bones are easily palpable), and 1 = emaciated mice (the skeletal structure is extremely prominent and the vertebrae are distinctly segmented). Body condition was recorded starting at age 43–79 d until mice reached CS 5.

### Clinical Score

Using an 8-point scale, clinical score measurements for mice in the 4 intervention studies started at age 50–81 d until mice reached CS 5. The clinical score was based on signs exhibited by the mice to identify the severity of the disease: 0 = no evidence of disease, 1 = shaking or splaying of the hindlimbs when suspended by the tail (an indication of weakness in the hindlimbs), 1.5 = weakness in one hindlimb (compensation for footdrop), 2 = change in gait (used as disease onset when attained on two consecutive days), 2.5 = extreme weakness in one hindlimb (inability to dorsiflex), 3 = extreme weakness in both hindlimbs, 3.5 = functional paralysis in one hindlimb, 4 = functional paralysis in both hindlimbs but can right themselves in less than 20 s after being placed on their side, and 5 = cannot right themselves to sternum within 20 s after being placed on their sides (endpoint).

### Statistical Analysis

Data for all 162 mice were submitted to a one-way repeated measures ANOVA to determine significant differences between CS4, CS4+ and CS5. When ANOVA indicated significance, a Tukey's HSD post hoc was used to determine the source of difference. A Pearson product-moment correlation coefficient (r) was determined to establish the relationship between the different endpoints. A Bland-Altman plot was used to analyze the agreement between the different endpoints. A logrank test was used to determine whether there was a difference in the rate at which mice reached CS4, CS4+ and CS 5. For all logrank tests, CS 4 was used as the reference when comparing CS 4 vs. CS 4+ and CS 4 vs. CS 5, whereas CS 4+ was used as the reference when comparing CS 4+ vs. CS 5. All ANOVA, linear regression and logrank test comparisons were planned. All statistical analyses were completed using GraphPad Prism (version 4.0, GraphPad Software, La Jolla, CA). Significance was established at P≤0.05. Data are presented as means ± SD, unless otherwise indicated.

## Results

Mice reached CS 4 at 123.9±10.3 d, CS 4+ at 126.6±9.8 d and CS 5 at 127.6±9.8 d (Table 1). There was a significant main effect between endpoints (P<0.001), all being significantly different from each other (P<0.001 for all).

**Figure 1:**
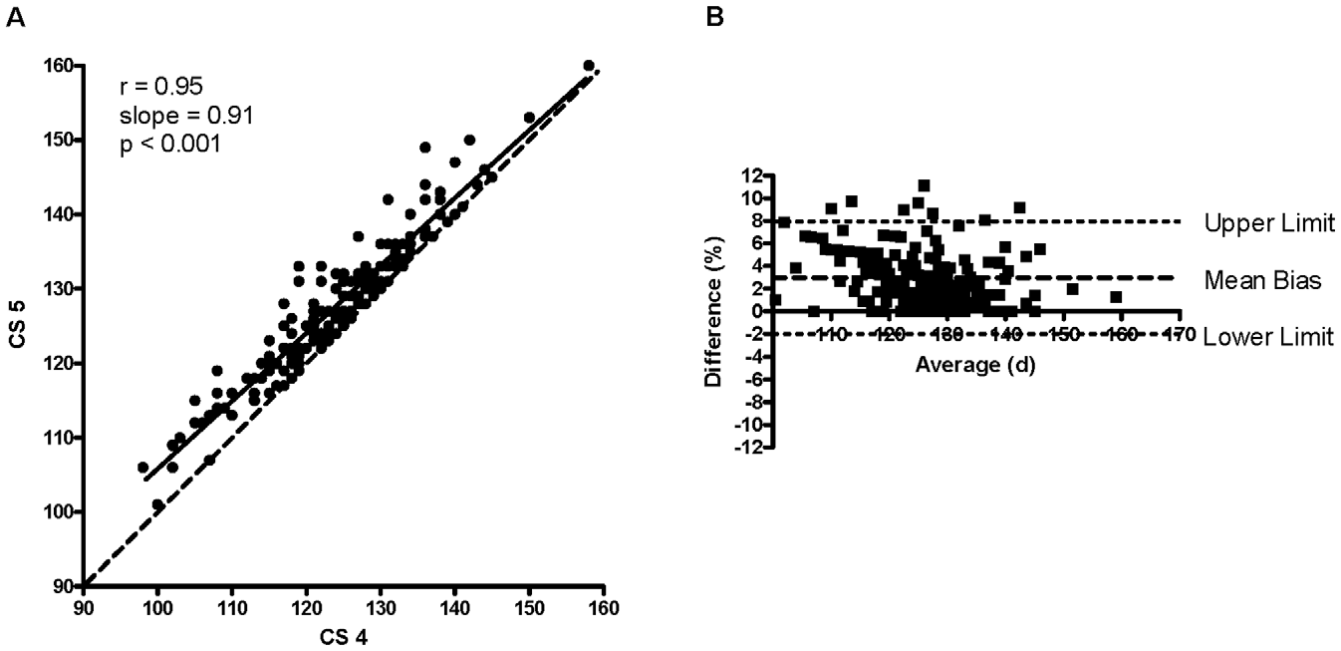
Correlation between CS 4 and CS 5 and the Bland-Altman plot for CS 4 vs. CS 5. (A) Correlation between CS 4 (clinical score of 4 = functional paralysis of both hindlimbs) and CS 5 (clinical score of 5 = CS 4 plus a righting reflex >20 s). There was a strong positive relationship between CS 4 and CS 5 (r = 0.95, slope = 0.91, P<0.001). CS 5 (d) = (14.80±2.83)+[(0.91±0.02)×(CS 4 in d)], mean ± SEM. Dashed line indicates line of identity. (B) A Bland-Altman plot comparing CS 4 to CS 5. Mean bias ± SD = 3.0±2.5%, lower limit = −2.0%, upper limit = 7.9%.

**Figure 2:**
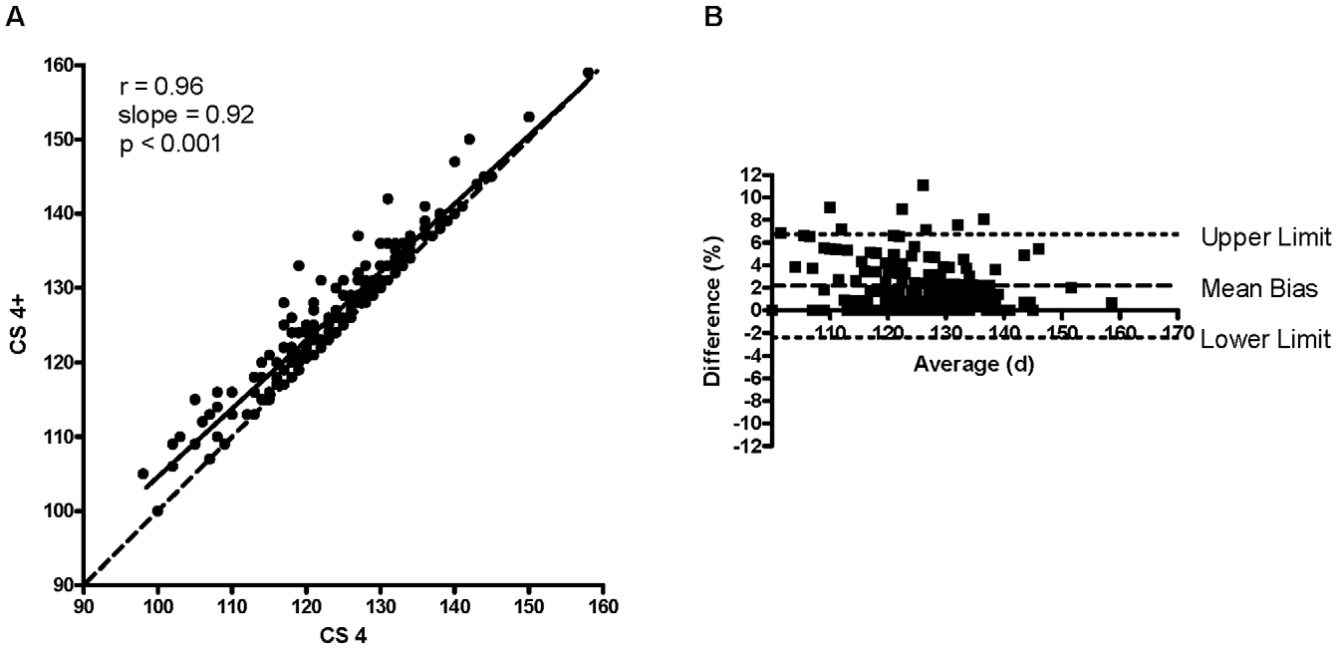
Correlation between CS 4 and CS 4+ and the Bland-Altman plot for CS 4 and CS 4+. (A) Correlation between CS 4 (clinical score of 4 = functional paralysis of both hindlimbs) and CS 4+ [CS 4 plus the earliest of a) weight loss ≥20% *vs.* body weight immediately prior to a clinical score of 2, b) weight loss ≥20% *vs.* peak body weight, c) body condition score <2, or d) a righting reflex >20 s (CS 5)]. There was a strong positive relationship between CS 4 and CS 4+ (r = 0.96, slope = 0.92, P<0.001). CS 4+ (d) = (12.79±2.56)+[(0.92±0.02)×(CS 4 in d)], mean ± SEM. Dashed line indicates line of identity. (B) A Bland-Altman plot comparing CS 4 to CS 4+. Mean bias ± SD = 2.2±2.3%, lower limit = −2.4%, upper limit = 6.7%.

**Figure 3:**
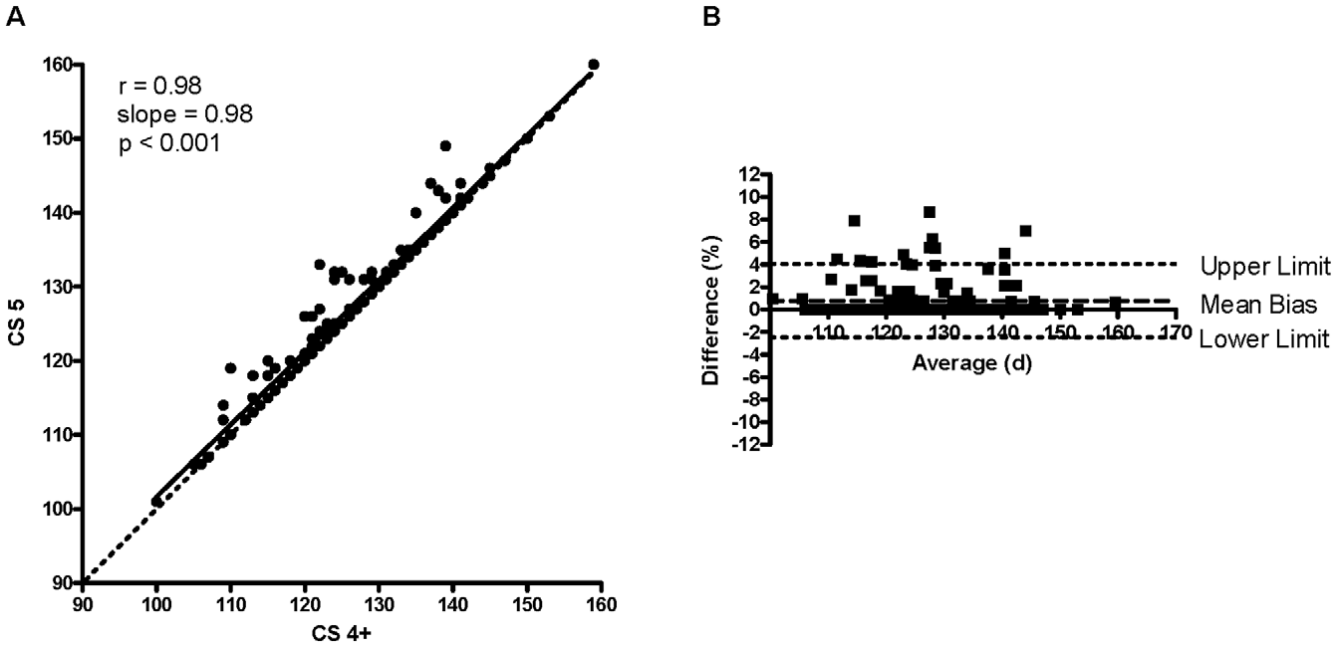
Correlation between CS 5 and CS 4+ and the Bland-Altman plot for CS 5 and CS 4+. (A) Correlation between CS 5 (clinical score of 5 = CS 4 and righting reflex >20 s) and CS 4+ [CS 4 plus the earliest of a) weight loss ≥20% *vs.* body weight immediately prior to a clinical score of 2, b) weight loss ≥20% loss *vs.* peak body weight, c) body condition score <2, or d) a righting reflex >20 s (CS 5)]. There was a strong positive relationship between CS 5 and CS 4+ (r = 0.98, slope = 0.98, P<0.001). CS5 (d) = (3.93±2.15)+[(0.98±0.02)×(CS 4+ in d)], mean ± SEM. Dashed line indicates line of identity. (B) A Bland-Altman plot comparing CS 5 to CS 4+. Mean bias ± SD = 0.8±1.7%, lower limit = −2.5%, upper limit = 4.1%.

There was a strong positive correlation between CS 4 and CS 5 (r = 0.95; slope = 0.91; P<0.001; Figure 1A), CS 4 and CS 4+ (r = 0.96; slope = 0.92; P<0.001; Figure 2A), and CS 4+ and CS 5 (r = 0.98; slope = 0.98; P<0.001; Figure 3A). The Bland-Altman plot revealed acceptable bias between CS 4 and CS 5 (3.0±2.5%; lower limit = −2.0%, upper limit = 7.9%; Figure 1B), between CS 4 and CS 4+ (2.2±2.3%; lower limit = −2.4%, upper limit = 6.7%; Figure 2B), and between CS 4+ and CS 5 (0.8±1.7%; lower limit = −2.5%, upper limit = 4.1%; Figure 3B).

**Figure 4:**
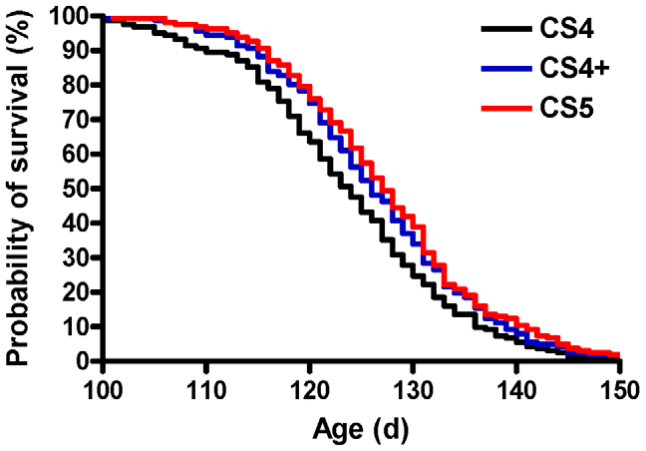
Probability of survival for CS 4, CS 4+ and CS 5. Probability of survival for the 3 different endpoints (CS 4, black line; CS 4+, blue line; CS 5, red line). For all logrank tests, CS 4 was used as the reference when comparing CS 4 vs. CS 4+ and CS 4 vs. CS 5, whereas CS 4+ was used as the reference when comparing CS 4+ vs. CS 5. The rate of reaching endpoint is significantly different (P = 0.021) between CS 4 (clinical score of 4 = functional paralysis of both hindlimbs), CS 4+ [CS 4 plus the earliest of a) weight loss ≥20% *vs.* body weight immediately prior to a clinical score of 2, b) weight loss ≥20% *vs.* peak body weight, c) body condition score <2, or d) a righting reflex >20 s (CS 5)], and CS 5 (clinical score of 5 = CS 4 and righting reflex >20 s). Mice reached CS 4 at a rate of 34% faster vs. CS 5 (HR = 1.34; 95% CI 1.10, 1.74; P = 0.006) and 24% faster vs. CS 4+ (HR = 1.24; 95% CI 1.00, 1.59; P = 0.046). Mice reached CS 4+ at a non-significant rate of 9% faster vs. CS 5 (HR = 1.09; 95% CI 0.88, 1.38; P = 0.410).

A logrank test showed a significant difference in the rate at which endpoint was reached between CS 4, CS 4+ and CS 5 (P = 0.021; Figure 4). Mice reached CS 4 at a rate 34% faster *vs.* CS 5 (HR = 1.34; 95% CI 1.10, 1.74; P = 0.006) and 24% faster *vs.* CS 4+ (HR = 1.24; 95% CI 1.00, 1.59; P = 0.046). Mice reached CS 4+ at a non-significant rate of 9% faster *vs.* CS 5 (HR = 1.09; 95% CI 0.88, 1.38; P = 0.410).

Statistical analyses were conducted for the same 3 endpoints within each sex. Differences between endpoints within each sex were similar as above.

## Discussion

Our objective was to determine whether an earlier endpoint could replace the most commonly used righting reflex in a transgenic mouse model of ALS. This was done to validate the use of an endpoint that would meet the strict standards set by research ethics boards to decrease suffering and distress in mice, as well as to allow researchers from different laboratories the use of a uniform and consistent endpoint to directly compare the effectiveness of treatments in this particular animal model. We found strong positive correlations between all endpoints with an acceptable mean bias as measured by a Bland- Altman plot. Using CS 4+ and CS 5 would prolong life span by 2% and 3%, respectively, as compared to CS 4. Additionally, mice reached CS 4 at a rate 24% faster compared to CS 4+ and 34% faster compared to CS 5.

Once mice reach CS 4, they must rely on the strength of their forelimbs to obtain food and water which may place them at risk of starvation and dehydration [43]. Some studies have used the inability to scrounge for food and water as endpoint [11], [13], [28], [43], [63], [67], [73], [106], however establishing this is time consuming and indicates an advanced disease state possibly well beyond CS 5. As well, research ethics committees may institute policy requiring mice to have access to food and water-based gels at cage floor level when mice reach a pre-defined disease severity, which actually prolongs disease exposure of mice due to easier access to nutrients.

Paw grip endurance and motor performance scores will have decreased precipitously by the time mice reached CS 4, as compared to scores prior to disease onset, due to hindlimb paralysis and weakness in the forelimbs [15], [82], [83]. A decrease in motor performance and/or paw grip strength has been previously used as endpoint in mouse models of ALS [3], [20]. Adoption of such endpoints requires expensive, specialized equipment such as the rotarod apparatus [20] and commercial grip strength meters [3]. Also, there is no standardization for the percent decrease in paw grip strength among laboratories using this as a criterion for endpoint [3], [20].

All mice in our analyses met the criteria for CS 4+, however when each additional criterion was assessed in isolation from CS 4, that is, as standalone criterion for endpoint (data collection ended at CS 5), only 29% of mice lost greater than 20% body weight versus their weight immediately prior to disease onset (20%CS2), 43% lost greater than 20% body weight versus peak weight (20%Peak), 26% had a body condition score of less than 2 (BC<2), while 100% met the criteria for CS 5 (Table 1). These results suggest that studies using any one of 20%CS2, 20%Peak, or BC<2 as a standalone criterion to establish endpoint would be keeping at least 57% of their mice alive past CS 5, prolonging disease exposure beyond what is considered humane. Alternatively, another interpretation of these results is that mice could die past CS 5 without meeting the standalone criteria 20%CS2, 20%Peak, or BC<2. Past CS 5, motor neuron degeneration is so far advanced that mice can no longer right themselves to scrounge for food and water and would be at a pronounced risk of starvation and dehydration. Our analyses also reveal that fewer than 9% of all mice met the standalone criteria 20%CS2, 20%Peak, or BC<2 prior to reaching CS 4 (Table 2). Hence, we conclude that CS 4 does not prolong disease exposure compared to 20%CS2, 20%Peak, or BC<2 in a mouse model of ALS. More male mice met 20%CS2, 20%Peak, or BC<2 prior to reaching CS 4 compared to females, however this result is expected since male mice have greater muscle mass than females and muscle atrophy is a result of disease progression [107].

**Table 2:** Raw data and summary of 162 B6SJL-TgN-(SOD1-G93A)1Gur autosomal hemizygous female (F) and male (M) mice meeting the additional endpoint criteria prior to reaching CS 4.

Endpoint Criteria	N Prior to CS 4 (F, M)	% of Mice Meeting Criteria Prior to CS 4 (F, M)	Mean Age (d) (F, M)
20%CS2	1 (0, 1)	0.6% (0%, 1.6%)	106.0 (NA, 106.0)
20%Peak	7 (3, 4)	4.3% (3%, 6.5%)	119.9±11.0 (123.7±15.2, 117.0±8.0)
BC<2	9 (1, 8)	5.6% (1%, 12.9%)	110.6±15.7 (108.0, 110.9±16.8)
Any of 20%CS2, 20%Peak or BC<2*	14 (3, 11)	8.6% (3%, 17.7%)	115.6±15.0 (123.0±16.1, 113.6±14.8)

20%CS2, weight loss ≥20% *vs.* body weight immediately prior to a clinical score of 2; 20%Peak, weight loss ≥20% *vs.* peak body weight; BC<2, body condition score <2.

*Earliest age (d) of 20%CS2, 20%Peak and BC<2 was used to calculate mean age. Data for Mean Age (d) are presented as means ± SD.

The righting reflex, either as a standalone criterion or used in conjunction with other parameters, has long been used to establish endpoint in a mouse model of ALS [1], [4], [5], [7], [10]–[15], [17]–[23], [25]–[28], [30]–[32], [36], [38]–[47], [49]–[51], [53], [54], [57]–[62], [64], [65], [68], [70]–[74], [77]–[91], [93]–[97]. The righting reflex has its advantages. Failure to right within a pre-defined period of time (at least >3 s) demonstrates severe muscle weakness, an indication of advanced motor neuron degeneration, as mice must use their strength to right themselves when placed on their side. The righting reflex may also be a measure of declining proprioception. Evidence suggests the dorsal root [102], dorsal root ganglia [102], [108]–[110], dorsal funiculus [102], Clarke's nuclei [103], [109], [111], [112] and spinocerebellar tract [103], [109], [111]–[113], the regions of the spinal cord responsible for processing proprioception, may be affected in humans with ALS [103], [108]–[112] and animal models of ALS [102], [113], however some researchers failed to ascertain this association [113]. It is important to note that the magnitude of diminished proprioception and muscle loss may be different depending on the time used as the cutoff for the righting reflex, with greater atrophy of motor neurons occurring when longer cutoffs are used [113]. Although the righting reflex may provide insight into muscle wasting and proprioception deficits, no studies have used the righting reflex to quantify proprioception and motor neuron loss. Rather, the righting reflex is simply used to identify endpoint. Moreover, the time used to establish the righting reflex is not standardized (at least 3 s), introducing a confounding within the righting reflex methodology. Some studies did not specify the length of time used as the cutoff for the righting reflex [26], [54], [60], [73], [78], [85], [90], [93].

The criteria for the ideal endpoint would meet strict ethical standards, be easily adopted by research laboratories, and ensure researchers are able to gather information regarding the progression of ALS and the effectiveness of treatments in intervention studies. Also, it would represent a point in the progression of the disease beyond which additional insight into the nature of disease or into the effectiveness of an intervention is absent, or at least nominal. CS 4, representing functional paralysis in both hindlimbs, occurs in all mice used in mouse models of ALS. CS 4 is reached earlier than both CS 4+ and CS 5, satisfying standards set by research ethics committees by shortening the time of disease exposure. A more difficult challenge arises when addressing the final criterion required to establish an ideal endpoint, that is, does CS 4 permit investigators the acquisition of sufficient data relating to disease progression? In rodent models of ALS, functional paralysis marks the beginning of the end in disease progression. Once paralysis is established in the hindlimbs, it will spread to the diaphragm, ultimately resulting in death due to respiratory failure. Between disease onset and hindlimb paralysis, changes in gait, paw grip strength and endurance, and motor performance deteriorate measurably allowing scientists to track these changes throughout the course of the disease [15], [82], [83]. These changes continue to occur past CS 4, but in severely disabled mice with compromised quality of life. Our analysis has yielded an equation that will allow researchers to predict the age at which CS 4+ and/or CS 5 are attained, on average, from CS 4. Animal models of multiple sclerosis (experimental autoimmune encephalomyelitis; EAE) [114]–[116] follow a similar disease progression, including hindlimb weakness and paralysis, and use similar endpoint criteria as mouse models of ALS, suggesting our findings may be used in mouse models of EAE.

Is CS 4 the “ideal” endpoint? The righting reflex may provide information regarding muscle loss and proprioception deficits beyond that of CS 4. For those specific studies whereby severe muscle loss and compromised proprioception are inherent outcome measures reflecting the effectiveness of a specific intervention, CS 5 should be adopted as an endpoint. Alternatively, we have shown that CS 4, occurring on average 4 days sooner than CS 5, can predict the age at CS 4+ or CS 5. These 4 days will lessen the suffering and distress experienced by mice used in mouse models of ALS. Adopting CS 4 as endpoint negotiates an acceptable agreement between scientific discovery and ethics, a partnership that serves to protect scientific integrity and ethical standards in the humane treatment of research animals. At the forefront is the strength of the data extrapolated from animal-based research to serve as the background for potential recommendations adopted for people with ALS.
